# Genomic Epidemiology of SARS-CoV-2 in Urban Settings in Senegal

**DOI:** 10.3390/v15061233

**Published:** 2023-05-24

**Authors:** Anna Julienne Selbé Ndiaye, Mamadou Beye, Gora Lo, Idir Kacel, Aissatou Sow, Nafissatou Leye, Abdou Padane, Aminata Mboup, Halimatou Diop-Ndiaye, Cheikh Sokhna, Coumba Touré Kane, Philippe Colson, Florence Fenollar, Souleymane Mboup, Pierre-Edouard Fournier

**Affiliations:** 1 Institut de Recherche en Santé, de Surveillance Epidémiologique et de Formation, Dakar 7325, Senegal; 2 IHU-Méditerranée Infection, 19-21 boulevard Jean Moulin, 13005 Marseille, France; 3 Laboratoire Bactériologie-Virologie, Hôpital Aristide Le Dantec, Dakar 3001, Senegal; 4 VITROME, Campus International IRD-UCAD de l’IRD, Dakar 1386, Senegal; 5 IRD, AP-HM, SSA, VITROME, Aix Marseille University, 13005 Marseille, France; 6 IRD, AP-HM, MEPHI, Aix Marseille University, 13005 Marseille, France

**Keywords:** Senegal, SARS-CoV-2, genomic, epidemiology, SNPs, COVIDSeq

## Abstract

We used whole genome sequencing to identify and analyze mutations in SARS-CoV-2 in urban settings during the deadliest wave of the COVID-19 epidemic—from March to April 2021—in Senegal. Nasopharyngeal samples testing positive for SARS-CoV-2 were sequenced on the Illumina NovaSeq 6000 sequencing system using the COVIDSeq protocol. A total of 291 genotypable consensus genome sequences were obtained. Phylogenetic analyses grouped the genomes into 16 distinct PANGOLIN lineages. The major lineage was B.1.1.420, despite circulation of the Alpha variant of concern (VOC). A total of 1125 different SNPs, identified relative to the Wuhan reference genome, were detected. These included 13 SNPs in non-coding regions. An average density of 37.2 SNPs per 1000 nucleotides was found, with the highest density occurring in ORF10. This analysis allowed, for the first time, the detection of a Senegalese SARS-CoV-2 strain belonging to the P.1.14 (GR/20J, Gamma V3) sublineage of the Brazilian P.1 lineage (or Gamma VOC). Overall, our results highlight substantial SARS-CoV-2 diversification in Senegal during the study period.

## 1. Introduction

The study of human coronaviruses has seen renewed interest since the beginning of the 21st century, with the emergence of deadly pneumonia occurring as major epidemics in humans [1]. In 2003, a major pneumonia outbreak started in China and spread, within a few months, to 25 countries on five continents, causing 774 deaths (case fatality rate of 9.6%) [2]. The causative agent was a new coronavirus named Severe Acute Respiratory Syndrome Coronavirus (SARS-CoV) [3]. In 2012, another new coronavirus was isolated and characterized for the first time in the Middle East in a man who died from pneumonia and renal failure [4]. This Middle East respiratory syndrome coronavirus (MERS-CoV) was the cause of a second worldwide pneumonia epidemic associated with 35% lethality [5]. In December 2019, cases of pneumonia of unknown etiology occurring in a group of individuals in Wuhan, Hubei, China were reported to the World Health Organization (WHO) and rapidly spread worldwide [6]. Metagenomic analysis of next-generation sequencing (NGS) data derived from samples from patients with this pneumonia enabled the characterization of a novel coronavirus [7]. The genome of this new coronavirus, classified as a betacoronavirus, shares 96% identity with a bat coronavirus, 79% identity with SARS-CoV and 50% identity with MERS-CoV [7,8].

The advancement of NGS has played an important role in our understanding of the COVID-19 pandemic. Indeed, viral metagenomic next-generation sequencing enabled the deciphering of the first complete SARS-CoV-2 genome detected in China; this complete sequence was publicly shared on 10 January 2020 [9]. The SARS-CoV-2 reference genome consists of 29,870 nucleotides encoding 9744 amino acids [10].

In Africa, the first SARS-CoV-2 genome sequence to be made publicly available and shared in a phylogenetic study was from a Nigerian index case [11]. In Senegal, the first four complete SARS-CoV-2 genome sequences were obtained from the first four positive cases at the Pasteur Institute in Dakar. All sequences are deposited in the Global Influenza Data Sharing Initiative (GISAID) database under accession numbers EPI_ISL_418206–9 [12]. Subsequently, genomic surveillance of SARS-CoV-2 in Senegal was intensified to monitor the evolution of the virus. This led to the identification of several VOCs [13]. A study that focused on the mutational analysis of the Spike protein in 2020–2021 revealed new combinations of mutations and evidence of local diversification [14]. Significant lineage diversification was also reported by another study during the first and second waves of the epidemic [15]. A comprehensive molecular characterization of the number and distribution of SNPs across the entire genome of Senegalese SARS-CoV-2 strains has been poorly explored. This study was conducted using amplicon-based sequencing following the ARTIC protocol; sequencing based on a metagenomic approach using probe enrichment (xGen) and focused on spike protein mutations was also conducted.

Prior to the present study, there were little data on COVID-19 from urban areas of Senegal. Thus, by applying the Illumina COVIDSeq protocol to Senegalese samples for the first time, we aimed to unveil the genetic epidemiology of Senegalese SARS-CoV-2 strains using nasopharyngeal samples collected in an urban environment during the second wave of the epidemic—which was considered the most deadly in Senegal, with a case-fatality rate of 3.16% [16]—through SNP characterization and analysis of the phylogenetic distribution.

## 2. Materials and Methods

### 2.1. Sampling

For this genomic study, we used a retrospective panel of 379 respiratory specimens (nasopharyngeal and oropharyngeal) that initially tested positive for SARS-CoV-2 based on diagnostic Real-Time Polymerase Chain Reaction (RT-PCR) conducted using various commercial kits selected based on their availability during the epidemic (Table 1). RT-PCR assays were performed on the molecular biology platform of the Institut de Recherche, en Santé, de Surveillance Epidémiologique et de Formation (IRESSEF), Diamniadio, Senegal. These samples were obtained mainly from patients living in the regions of Dakar and Thiès, which are the two cities in Senegal that were most affected by COVID-19 [17]. The samples were collected from suspected cases, contact cases of confirmed COVID-19 cases and travelers leaving the Senegalese territory. These samples, selected for genome sequencing using the Illumina COVIDSeq protocol, had cycle threshold (Ct) values of between 15 and 30. The samples were transported in biohazard containers (DHL Company, Dakar, Senegal) to the Institut Hospitalo-Universitaire Méditerranée Infection-IHUMI (Marseille, France). Of the 379 samples, 352 were collected between March and April 2021, which coincided with the second and most deadly wave of the epidemic in Senegal. To document the genetic diversity in the first wave—during which genomic surveillance was not effectively conducted in Senegal—27 samples collected between June and July 2020 were included among the sequenced samples.

### 2.2. Isolation of Viral RNA

For samples selected for sequencing, a new round of RNA extraction from the primary sample was performed. The MagMAX^TM^ Viral/Pathogen II Nucleic Acid Isolation kit (Thermofisher Scientific, Vilnius, Lithuania) was used to extract viral RNA on a King Fisher instrument (Thermo Scientific™ 5400620). Based on the manufacturer’s instructions, viral RNA was extracted from 200 µL of the primary sample and eluted in 80 µL of elution buffer. The extracted RNA was stored at −80 °C until sequencing.

### 2.3. Preparation and Sequencing of Libraries

Manual preparation of sequencing libraries was performed following the Illumina COVIDSeq test protocol according to the manufacturer’s instructions (Illumina Inc., San Diego, CA, USA), as previously described [18].

Extracted RNA was annealed using random hexamers. First strand cDNA was then synthesized from the annealed RNA using a reverse transcriptase. After synthesis, the cDNA was amplified. For this amplification, two separate multiplex PCR reactions were performed with two primer pools using Illumina COVIDSeq test (3072 Samples, #20043675). This amplification produced 98 amplicons for SARS-CoV-2 detection and 11 amplicons targeting the human genome for internal control. The amplicon sets from the two PCRs were pooled. One COVIDSeq Positive Control HT (CPC HT) and one No Template Control (NTC) were placed in each 96-well plate.

Following cDNA synthesis and amplification, PCR amplicons were fragmented and tagged with adapter sequences. The tagged amplicons were purified according to the manufacturer’s instructions (Illumina Inc., San Diego, CA, USA). For the addition of indexes to the tagged amplicons and sequences required for the sequencing cluster, amplification of the tagged amplicons was performed using IDT^®^ for Illumina-PCR Indexes Set 1–4 (384 indexes, #20043137). Libraries were subsequently pooled and purified based on the manufacturer’s guidelines (Illumina Inc., San Diego, CA, USA). Each library pool contained 96 libraries from one plate (94 samples, one CPC and one NTC).

Each library pool was quantified and then normalized. For quantification, 2 ul of each library pool was analyzed on the Qubit 4.0 fluorometer using the dsDNA HS Assay Qubit™ kit (ThermoFisher Scientific, MA USA). Based on this quantification, the molarity (nM) of each library pool was calculated by setting the average library size to 400 bp. Each library pool was then normalized to 4 nM. The normalized pools were pooled in a sequencing pool with a final loading concentration of 100 pM for a total of 384 samples (index sets 1–4). The sequencing pool was diluted to 0.5 nM using 10 mM Tris-HCl (pH 8.5) and then denatured using sodium hydroxide (0.2 N NaOH) and neutralized using 400 mM Tris-HCl (pH 8.5). Finally, the denatured library pool was loaded onto the SP Flow Cell following the NovaSeq-XP workflow according to the manufacturer’s guidelines (Illumina Inc.) and sequenced on the Illumina Novaseq 6000 sequencing system using sequencing by synthesis (SBS) chemistry.

### 2.4. Data Analysis

#### 2.4.1. Consensus Sequences

An in-house pipeline, inspired by the Illumina analysis pipeline but with some modifications, was used to assemble the Illumina sequencing data into consensus genomes [19]. The raw data were demultiplexed into fastq files. Using trimmomatic (v 0.39), we performed quality control of the fastq files and adaptor trimming of raw reads [20]. Minimap2 (version 2.24) was used to map the fastq files back to the SARS-CoV-2 reference genome indexed with GenBank accession no. NC_045512.2. Subsequently, the mapped genomes were converted to BAM files, sorted and indexed. Duplicate sequences and primers were removed using samtools (version 1.15.1). Using an in-house script written in the Python programming language, consensus genomes were generated using Sam2consensus (https://github.com/vbsreenu/Sam2Consensus) (accessed on 7 April 2022).

#### 2.4.2. Genomic and Phylogenetic Analyses

The obtained consensus whole genome sequences were mapped against the SARS-CoV-2 Wuhan-Hu-1/2019 reference genome sequence (Genbank: MN908947) and then analyzed using the Nextclade (v1.14.1) web application [21] for mutation detection and determination of genomic locations of SNPs. In addition, mutations were assessed using the CoVsurver mutations web application available on the GISAID website [22]. To construct the initial maximum likelihood phylogenetic tree, the obtained genomic sequences were aligned against the reference sequence of SARS-CoV-2 (GenBank accession no. NC_045512.2) using a multiple sequence alignment program (MAFFT v7.505). From the obtained alignment, a phylogenetic tree was reconstructed using IQ-TREE2 with the GTR+I+G model and 1000 ultrafast bootstrap repeats. The generated tree was stored in treefile format. This file was then uploaded to the online software Interactive Tree Of Life (iTOL) [23] for tree visualization and annotation. For genetic lineage assignment, we used the two commonly used dynamic classification systems: Nextclade web tool (v1.14.1) and Phylogenetic Assignment of Named Global Outbreak Lineages (PANGOLIN) software v1.1.14 (https://github.com/hCoV-2019/pangolin (accessed on 15 July 2022) [24]. SNP distribution and density were determined using a set of Excel formulas packaged by us: Density (SNPs/1000 nt) = (Number of SNPs × 1000 nt) ÷ Length of genomic region. A second in-house Python script was used during the consensus sequence analysis to analyze the different SARS-CoV-2 genotypes obtained from these Senegalese SARS-CoV-2 sequences.

#### 2.4.3. 3D Structural Mapping of the Spike Protein

The 3D structure of the spike protein with the identified amino acid changes was constructed using the CoVsurver tool [22]. The fasta sequences were submitted to the *CoVsurver: Mutation Analysis of hCoV-19* web application and the major mutation sites in the S protein were analyzed and annotated using hCoV-19/Wuhan/WIV04/2019 as the reference strain. The 3D image of the spike protein obtained from the web application was saved.

#### 2.4.4. Ethical Statement

This study received ethical approval from the National Health Research Ethics Committee of Senegal (opinion 000159/MSAS/CNERS/Sec, 21 August 2020). The samples were collected with the free and informed consent of each study participant.

## 3. Results

### 3.1. Characteristics of the Study Population

Of the 379 sequenced samples, 75% (284/379) were from the Thiès region while 25% (95/379) were from the Dakar region. The mean age of the patients was 49 years (3–98 years). The male/female ratio was 1.51 (227/150). Information on gender was not available for two patients. The samples used in this study were sampled prior to the introduction of COVID-19 vaccination in the Senegalese population.

### 3.2. Lineage Distribution, Clades, and Phylogeny

**Figure 1:**
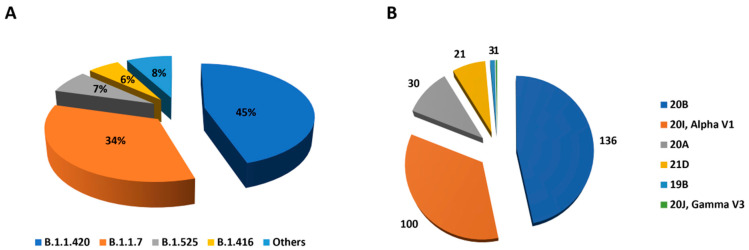
Distribution of the different clades and lineages of SARS-CoV-2 in the Dakar and Thiès regions of Senegal. (**A**) Distribution of 291 genotypable individuals into 16 lineages according to the PANGOLIN (Phylogenetic Assignment of Named Global Outbreak Lineages) classification. Lineage B.1.1.420 was the major lineage (131/291, 45%,), followed by lineage B.1.1.7 (100/291, 34%), B.1.525 (21/291, 7%) and B.1.416 (16/291, 6%). The remaining lineages (A.27, B.1, B.1.160, B.1.617, B.1.629, B.1.1, B.1.1.318, P.2, R.1 and P.1.14) represented 8% of the sequences. (**B**) Distribution of the 291 genotypable sequences into six Nextstain clades using the Nextclade web tool. Clade 20B was predominant (136/291), followed by clade 20I, Alpha V1 (100/291) and clade 20A (30/291). Clades 21D, 19B and 20J Gamma V3 contained 21, 3 and 1 of the genotypable sequences, respectively.

**Figure 2:**
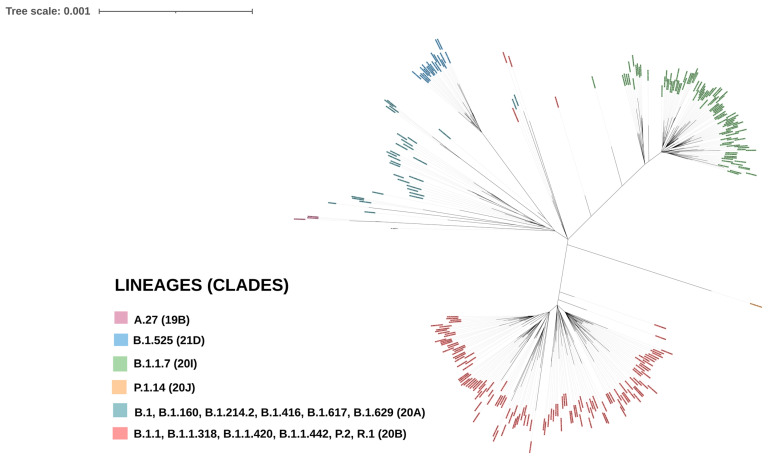
Maximum likelihood phylogenetic tree of the 291 whole genome sequences of Senegalese SARS-CoV-2 strains. The tree shows the clades circulating in Senegal during the second wave of the epidemic. The 291 sequences are grouped into six clades based on the Nextstrain nomenclature. Clade 19B includes strains of the PANGOLIN A.27 lineage; Clade 20A includes strains from PANGOLIN lineages B.1, B.1.160, B.1.214.2, B.1.416, B.1.617 and B.1.629; strains of PANGOLIN lineages B.1.1, B.1.1.318, B.1.1.420, B.1.1.442, P.2 and R.1 are grouped in clade 20B; clade 20I contains strains of PANGOLIN lineage B.1.1.7; clade 20J contains the single isolate of PANGOLIN lineage P.1.14; strains of PANGOLIN lineage B.1.525 are grouped in clade 21D.

The median number of raw reads generated from the 379 sequenced strains was 2,961,812, with a minimum of 254,768 reads and a maximum of 6,712,590 reads (Supplementary Data S4). After genomic analysis, all sequences with more than 50% missing nucleotides were excluded from analysis. In total, 291 genotypable sequences were obtained from the 379 sequenced samples. These sequences had an average depth of 16,163X (403X–48,157X) and an average genome coverage of 92% (Genomic coverage range of 50–100%). For each sample, the total number of raw reads, total number of mapped reads, sequencing depth and coverage are listed in Supplementary Data S4. Following quality control, 291 genomes, including 12 strains collected in July 2020 and 279 strains collected in March and April 2021, were included in the analysis. The 291 genomes have been deposited in the public GISAID database (accession nos. EPI_ISL_13307473–557, EPI_ISL_13710664–722, EPI_ISL_16941819–97, EPI_ISL_16942089–145, EPI_ISL_16987269–76, EPI_ISL_1827867, EPI_ISL_1855408, EPI_ISL_13325688 and EPI_ISL_16941818) (Supplementary Data S1). These sequences were then subjected to phylogenetic analysis. The 291 sequences were grouped, according to the PANGOLIN classification, into 16 SARS-CoV-2 lineages (Figure 1A). Of these, B.1.1.420 was the major lineage (131/291, 45.01%) followed by B.1.1.7 (100/291, 34.36%), B.1.525 (21/291, 7.21%) and B.1.416 (16/291, 5.49%). The 23 other sequences were grouped into the following other lineages: A.27 (n = 3), B.1 (n = 7), B.1.160 (n = 1), B.1.214.2 (n = 4), B.1.617 (n = 1), B.1.629 (n = 1), B.1.1 (n = 1), B.1.1.318 (n = 1), B.1.1.442 (n = 1), P.1.14 (n = 1), P.2 (n = 1) and R.1 (n = 1). The B.1.416 lineage was found in the Thiès region only. Additionally, we assigned the genomes to Nextstrain clades using the Nextclade web tool. The analyzed genomes were grouped into six distinct clades: 19B, 20A, 20B, 20I (Alpha, VI), 20J (Gamma, V3) and 21D. The 20B clade, correlated with the B.1.1.420 lineage, was predominant (136/291, 46.73%), followed by the 20I (or Alpha) clade correlated with the B.1.1.7 lineage (100/291, 34.36%) and the 20A clade correlated predominantly with the B.1.416 lineage (30/291, 10.3%) (Figure 1B). The initial maximum likelihood phylogenetic tree constructed with the 291 sequences and rooted with the Wuhan reference sequence indexed with GenBank accession no. NC_045512.2 confirmed the clustering of the analyzed sequences into these main groups (Figure 2).

### 3.3. Mutation Analysis

**Figure 3:**
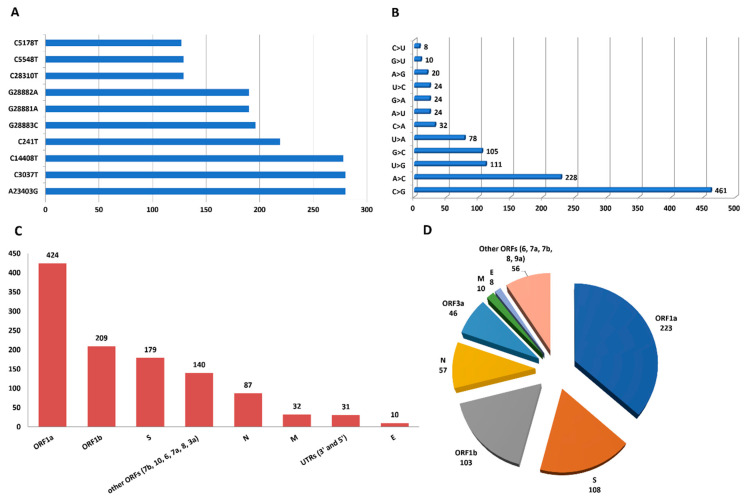
Representation of the mutational analysis of 291 whole genome sequences of Senegalese SARS-CoV-2. (**A**) Representation of the 10 most frequently detected SNPs (Single Nucleotide Polymorphisms): A23403G, C3037T, C14408T, C241T, G28883C, G28881A, G28882A, C28310T, C5548T and C5178T. (**B**) Representation of the 10 most frequent nucleotide substitutions. Among the 1125 SNPs, the following 10 nucleotide substitutions were the most common: C>U transversion, G>U transversion, A>G transition, U>C transversion, G>A transversion, A>U transversion, G>C transversion, U>A transversion, C>A transversion, U>G transversion, A>C transversion and C>G transversion. (**C**) Distribution of SNPs in different regions of the Senegalese SARS-CoV-2 genomes. The greatest number of SNPs was counted in ORF1a, followed by ORF1b and the S gene. A total of 140 SNPs were counted in the other ORFs (Open Reading Frames). A total of 87, 32 and 10 SNPs were counted in the genes coding for the N, M and E proteins, respectively. A total of 31 SNPs were identified in the 5′ and 3′ UTRs (UnTranslated Regions) of the Senegalese SARS-CoV-2 genomes. (**D**) Distribution of non-synonymous mutations across different regions of the genomes. Non-synonymous mutations were found in all coding regions of the Senegalese SARS-CoV-2 genomes. The largest number of non-synonymous mutations was found in ORF1a while the smallest number was found in ORF6.

A total of 1125 SNPs, including 13 SNPs in non-coding regions, were identified across the 291 genotypable sequences with respect to the reference NC_045512.2 sequence. Of the 1125 SNPs, 718 were singleton SNPs i.e., detected only once, while 407 were informative SNPs (Supplementary Data S2). The 10 most frequently detected SNPs were A23403G, C3037T, C14408T, C241T, G28883C, G28881A, G28882A, C28310T, C5548T and C5178T (Figure 3A). They were found in 280, 280, 278, 219, 196, 190, 190, 129, 129 and 127 of the sequences, respectively. The complete list of all SNPs is available in the supplementary data (Supplementary Data S2). The C>U transition was the most common (461/1125, 41%), followed by the G>U transversion (228/1125, 20%) and the A>G transition (111/1125, 10%) (Figure 3B). Other substitutions were C>G (8/1125, 0.7%), A>C (10/1125, 0.8%), U>G (20/1125, 1.7%), G>C (24/1125, 2.1%), U>A (24/1125, 2.1%), C>A (24/1125, 2.1%), A>U (32/1125, 2.8%), G>A (78/1125, 6.9%) and U>C (105/1125, 9.3%).

A total of 487 SNPs were identified in the main B.1.420 lineage, while 335 and 87 SNPs were counted in the B.1.1.7 (100/291) and B.1.525 (21/291) lineages, respectively. Among the amino acid substitutions that occurred in lineage B.1.1.7 sequences, the presence of the Q27* mutation in ORF8, which changes a CAA sense codon to a UAA stop codon, was noted. An average density of 37.2 SNPs per 1000 nucleotides (NTs) was found in the studied SARS-CoV-2 genomes. The highest SNP density per 1000 NTs was found in ORF10 (85.5 SNPs/1000 NTs) while the lowest was found in ORF1b (25.8 SNPs/1000 NTs) (Table 2). The highest number of SNPs was found in ORF1a (424/1125, 38%), followed by ORF1b (209/1125, 19%) and the S gene (179/1125, 16%) (Figure 3C).

These single nucleotide variations resulted in 611 non-synonymous mutations. The non-synonymous mutations occurred in the Spike protein (n = 108), envelope protein E (n = 8), membrane glycoprotein M (n = 10), N nucleocapsid phosphoprotein (n = 57), ORF1a (n = 223), ORF1b (n = 103), ORF3a (n = 46), ORF6 (n = 7), ORF7a (n = 8), ORF7b (n = 6), ORF8 (n = 24) and ORF9b (n = 11) (Figure 3D). The most frequent non-synonymous mutation (D614G) was driven by the SNP at position 23,403 changing adenosine to a guanosine (A23403G). This amino acid change occurred in 276/291 (94.8%) of the SARS-CoV-2 genomes. The single nucleotide variation at position 3037 (C3037T) resulted in an amino acid change (P314L) in ORF1b that was the second most common non-synonymous mutation in the analyzed sequences (257/291, 88.3%). A total of 35 amino acid deletions were also found. They occurred in ORF1a (n = 10), the spike protein (n = 7), ORF8 (n = 6), ORF3a (n = 4), ORF7b (n = 3), ORF7a (n = 2), the nucleocapsid protein (n = 2) and ORF6 (n = 1). Amino acid deletions F3677-, S3675- and G3676- caused by ORF1a nucleotide deletion Δ3675–3677; H69-/V70- caused by S deletion Δ21765–21770 and Y144- were the most frequent amino acid deletions detected here (Supplementary Data S2). No amino acid insertions were noted in these sequences.

### 3.4. Sequence Analysis of the SINGLE GAMMA SUBLINEAGE ISOLATE (P.1.14) from Senegal

**Figure 4:**
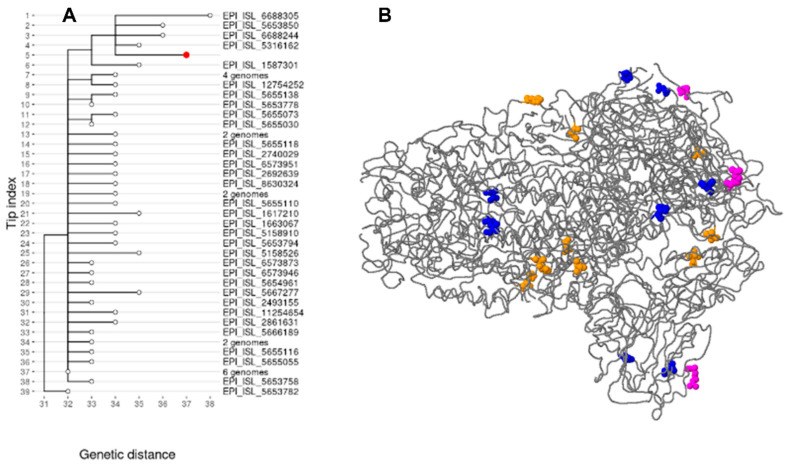
Representation of the Senegalese isolate of the Gamma sublineage (P.1.14). (**A**) Phylogenetic analysis of the Senegalese isolate P.1.14. The isolate was linked to 59 genomes classified as gamma variants of concern in the Global Initiative on Sharing Avian Influenza Data (GISAID) database. These gamma variants originated from the United States, Canada, Mexico, Argentina and Brazil. The Audacity Instant application in GISAID was used to generate a phylogenetic tree. Branch lengths are based on the genetic distance (the number of estimated mutations). Sequences in the tree are designated by their GISAID accession numbers or the number of genomes in the group. The Senegalese isolate P.1.14 is represented on the tree by a red dot. (**B**) 3D structural visualization of the spike glycoprotein of the Senegalese isolate P.1.14. The 10 amino acid changes identified in the Senegalese isolate in comparison with the Wuhan reference sequence are visualized by colored balls according to the following legend: non-synonymous mutations with a possible phenotypic effect are colored in yellow: L18F, K417T, D614G; non-synonymous mutations with an unknown effect are colored in pink and blue: T20N, P26S, D138Y, T1027I, P1162S, V1176F, H655Y.

During this study, we were able to identify and characterize, for the first time in Senegal, an isolate classified in the sublineage P.1.14 (GR/20J, Gamma V3), which belongs to the Brazilian lineage P.1 (Gamma variant). This isolate was detected in the western part of Senegal, in the region of Thiès, more precisely in the health district of Meckhé. The infected patient was a 66-year-old male whose sample was collected on 12 March 2021 during the second wave of the epidemic. As the study was performed retrospectively, investigation of the patient’s travels could not be conducted. Mutation assessment using Nextclade (v1.14.1) and CoVsurver mutation web applications on the GISAID website [21] showed that this P.1.14 isolate’s genome was characterized by 33 substitutions compared with the SARS-CoV-2 Wuhan-Hu-1/2019 (Genbank: MN908947) reference genome (Supplementary Data S3). The most frequent of these substitutions was the replacement of C with U, which occurred 12 times. These single nucleotide variations resulted in 22 non-synonymous amino acid mutations and 3 deletions distributed as follows: 3 non-synonymous mutations (P80R, R203K, G204R) in the nucleocapsid protein; 3 non-synonymous mutations (S1188L, K1795Q, S2947N) and 3 deletions (S3675-, G3676-, F3677-) in ORF1a; 3 non-synonymous mutations (P314L, E1264D, A2513S) in ORF1b; 1 non-synonymous mutation (S253P) in ORF3a; 1 non-synonymous mutation (E92K) in ORF8; 1 non-synonymous mutation (Q77E) in ORF9b; and 10 non-synonymous mutations (L18F, T20N, P26S, D138Y, K417T, D614G, H655Y, T1027I, P1162S, V1176F) in the spike protein. In addition to the spike protein mutations common to the P.1 lineage, this P.1.14 isolate’s genome also harbored the P1162S mutation in the spike protein. Of the 291 sequences analyzed, the P1162S mutation occurred only in the P.1.14 isolate. Compared with the source P.1 lineage, this sublineage was marked by an absence of the combined E484K and N501Y mutations. We marked the mutation sites on the P.1.14 isolate using 3D structural visualization of the Spike protein (Figure 4B). A search for the sequences to which the Senegalese P.1.14 isolate was most closely related was performed in the global EpiCov database on the GISAID platform. The results showed that this Senegalese isolate’s genome was linked to 59 genomes classified as VOC Gamma originating from the United States, Canada, Mexico, Argentina and Brazil. The maximum likelihood tree further showed that the Senegalese isolate was most closely related to an isolate from Toronto and available in GISAID under accession number EPI_ISL_5316162 (Figure 4A). The Senegalese isolate is available on GISAID under accession number EPI_ISL_16942137.

## 4. Discussion

SARS-CoV-2 infections have been a global health priority since December 2019. Due to its high contagiousness, SARS-CoV-2 has spread rapidly and due to its relatively high mutation rate, variations are likely to appear during each replication cycle [25]. Since its emergence, different sequencing protocols have been adopted to allow the study of SARS-CoV-2’s genetic diversity and evolution [26]. In Senegal, most SARS-CoV-2 genomes were sequenced using amplicon-based high-throughput sequencing following the ARTIC protocol [27]. The analysis of sequence data from these samples was mostly oriented towards evaluation of the circulating SARS-CoV-2 lineages. In the present study, the COVIDSeq sequencing protocol was applied to COVID-19-positive samples collected during the deadliest wave of the epidemic in Senegal. The COVIDSeq approach provided information on the genetic epidemiology of Senegalese SARS-CoV-2 isolates. The number of SNPs identified was high compared with that reported in other studies conducted in Senegal [14]. This analysis identified mutational hotspots in the Senegalese SARS-CoV-2 genome; the 3′ UTR, ORF10 and ORF3a regions were the top three mutational hotspots. ORF10 was the coding region with the highest SNP density. This is similar to the results obtained for SARS-CoV-2 genomes from Nigeria [28]; however, compared with our study, the study of Nigerian SARS-CoV-2 genomes found a lower number of SNPs, a difference likely caused by the sample size.

The phylogenetic analysis performed here provided insights into the dominant lineages circulating during the deadliest COVID-19 wave in Senegal. Lineage B.1.1.420 dominated the second wave, even though this period coincided with the circulation of the Alpha B.1.1.7 VOC. This B.1.1.420 predominance in Senegal during the second COVID-19 wave was also demonstrated by a study based on a Bayesian phylogeographic approach that showed that lineage B.1.1.420 was introduced into Senegal from Italy before the increase in its transmissibility and suggested that its predominance over the Alpha VOC may be associated with a fitness advantage [15]. Similarly, a study describing the dynamics of variants during epidemic waves in Senegal showed that lineage B.1.1.420 was among the predominant lineages in the second wave [27].

Analysis of SARS-CoV-2 genomes obtained in the present study showed that the most common non-synonymous mutations in SARS-CoV-2 strains from this deadly wave in Senegal was the D614G mutation, which occurred in a majority of the analyzed sequences. This mutation, associated with increased infectivity [29], first appeared in Senegal in December 2020 [13] and is one of the most prevalent mutations in African SARS-CoV-2 viruses [30]. The ORF1b-P314L mutation was the second most prevalent, occurring in almost one tenth of the analyzed genomes. The nearly systematic association between spike-D614G and ORF1b-P314L mutations in Senegalese SARS-CoV-2 strains has been reported in all geographical areas [31].

Regarding nucleotide substitutions across the SARS-CoV-2 genomes, we observed an abundance of the C>U transition, which occurred in 41% of the SARS-CoV-2 genomes. This is in agreement with findings from other studies conducted worldwide. For instance, a study of 11,183 SARS-CoV-2 genomes from different regions of the world found an abundance of the C>U transition [32]. It has been reported that in viruses, transition mutations are more frequent than transversion mutations [33], indicating a lower abundance of Cytosine-phosphate-Guanine (CpG) motifs resulting from Cytosine methylation and Uracil deamination. In fact, among all known betacoronaviruses, SARS-CoV-2 was reported to have the highest CpG deficiency and therefore more C>U transition mutations [34].

Remarkably, we detected, for the first time in Senegal, an isolate belonging to the P.1.14 sublineage. This isolate harbored most of the signature mutations of the Gamma VOC, which originated in Brazil [35]. Despite its high transmissibility and rapid local spread following its emergence, the Gamma variant had limited international transmission beyond Brazil [35]. By monitoring and evaluating the evolution of SARS-CoV-2, it has been shown that the Gamma variant has not become as established in Africa as it has on most other continents [36]. This rare introduction of the Gamma variant into West Africa was also demonstrated in another study in which only one sequence matching the Gamma VOC was detected among the 10,343 sequences collected during the second wave of the COVID-19 epidemic and submitted to GISAID [37]. As found in our study, three of the four African SARS-CoV-2 genomes classified as Gamma VOCs also lacked the E484K+N501Y combination mutation associated with high viral transmissibility [38]. This may explain the low circulation of the Gamma variant compared with other VOCs in Africa.

## 5. Conclusions

Our study has two limitations. The first one was the area of sample collection, which covered only two regions of Senegal, and the second one was the short study period. These limitations allowed for only a cross-sectional view of the genetic diversity of SARS-CoV-2 in Senegal. Nevertheless, this study provided a whole-genome mutational analysis of SARS-CoV-2 in urban areas of Senegal. Its results indicate the need for intensifying efforts for genomic surveillance of SARS-CoV-2 in Africa, although the availability of technical platforms can sometimes be limited. This might be conducted, including through implementation of variant-specific real-time PCR assays, to get an exhaustive picture of circulating variants and to target the most interesting genomes with NGS.

## Figures and Tables

**Table 1:** RT-PCR kits used for the diagnosis of COVID-19 in the samples included in this study.

Real-Time PCR System	Country	Regulation	Target Genes	Limit of Detection (copies/mL)	Thermal Cycler
Abbott RealTime SARS-CoV-2 assay (Abbott, Illinois, USA)	USA	US-IVD	RdRp et N	100	System m2000sp/rt
Allplex™2019-nCoV Assay (Seegene, Seoul, Republic of Korea)	Republic of Korea	CE-IVD	E, N, et RdRp	4167	Real-time PCR detection system CFX96 Touch, BIO-RAD
Detection Kit for 2019 Novel Coronavirus (2019-nCoV) RNA (PCR-Fluorescence Probing (DAAN GENE, Guangdong, China)	China	CE-IVD	ORF1ab et N	500	Amplix 16/Amplix NG48 system
Novel Coronavirus (2019-nCoV) Nucleic Acid Diagnostic Kit (Sansure Biotech, Hunan Province, China)	China	CE-IVD	ORF1ab et N	200	Real-time PCR detection system CFX96 Touch, BIO-RAD

E, envelope; N, nucleocapsid; RdRp, RNA dependent RNA polymerase; nCoV, novel coronavirus; USA, United States of America; US-IVD, United States-In Vitro Diagnostics; CE-IVD, European Certification; sp, sample preparation; rt, real-time; PCR, Polymerase Chain Reaction.

**Table 2:** Density and distribution of SNPs in Senegalese SARS-CoV-2 sequences.

	5′ UTR	ORF1a	ORF1b	S	ORF 3a	E	M	ORF 6	ORF7a	ORF 7b	ORF 8	N	ORF10	3′ UTR
Length (nt)	265	13,218	8088	3822	828	228	669	186	366	132	366	1260	117	229
Number of SNPs	9	424	209	179	64	10	32	12	19	7	28	87	10	22
SNP density (SNP/1000)	33.96	32.07	25.84	46.83	77.29	43.85	47.83	64.51	51.91	53.03	76.50	69.04	85.47	96.06

UTR, UnTranslated Region; ORF, Open Reading Frame; E, envelope; N, nucleocapsid; S, Spike; M, membrane glycoprotein; nt, nucleotides; SNP, Single Nucleotide Polymorphism.

## Data Availability

SARS-CoV-2 genomes analyzed in this study are available on the global initiative on sharing avian influenza data (GISAID) database (https://gisaid.org/(accessed on 16 February 2023).

## Supplementary Materials

The following supporting information can be downloaded at: https://www.mdpi.com/article/10.3390/v15061233/s1, Supplementary Data S1: metadata and accession numbers for sequences submitted to GISAID, Supplementary Data S2: complete list of SNPs, amino acid substitutions and amino acid deletions identified in Senegalese SARS-CoV-2 sequences, Supplementary Data S3: Complete list of SNPs, amino acid substitutions and amino acid deletions identified in the Senegalese isolate P.1.14 (Gamma, V3). Supplementary Data S4: reads data (raw and mapped) for each sequence.
